# The Fairytale Semantic Differential Technique: A Cross-Cultural Application

**DOI:** 10.3390/bs10070112

**Published:** 2020-07-06

**Authors:** Victor Petrenko, Olga Mitina

**Affiliations:** Department of Psychology, Lomonosov Moscow State University, Mokhovaya st. 11/9, 125009 Moscow, Russia; victor-petrenko@mail.ru

**Keywords:** psychosemantics, repertoire grids, Fairytale Semantic Differential, test of cognitive abilities, cognitive complexity, socialization, interpersonal perception, 4–10-year-old children, cross-cultural differences

## Abstract

The “Fairytale Semantic Differential” method, in which the respondent assesses several fairytale characters according to a set of personal characteristics, is designed for individual psychological work with children 4–10 years old. Personality characteristics, according to which the characters are evaluated, are formulated in terms understandable to child respondents, i.e., these are words that parents and teachers use when dealing with children of this age. An analysis of the child’s attitude to a certain fairytale character makes it possible to determine the individual properties of his/her moral value sphere. Quantitative indicators that can be calculated on the basis of the data collected by the questionnaire are discussed. These indicators characterize the child’s personality, his/her understanding of interpersonal relationships, the dimensionality of the categorial space of interpersonal perception, the content of these categories and its hierarchy, the level of cognitive development in this domain, and the degree of socialization. The results of an empirical study that was conducted in Moscow, Baku, and Tashkent are presented. Age and sex differences were found in the cognitive complexity of interpersonal perception and socialization. Also, an example of individual semantic space is presented.

## 1. Introduction

The psychosemantic approach has been used since the mid-1970s in the USSR and later in Russia as a methodology that allows research related to the reconstruction of people’s mentality [1,2]. This method was developed by combining the approaches of C. Osgood [3] and G. Kelly [4]. The Fairytale Semantic Differential (FSD) was designed by V. Petrenko [1] as a psychosemantic method to examine the cognitive complexity of children’s interpersonal perception and elicit personal constructs, as well as to determine their levels of self-esteem and socialization. This method was designed for children aged between 4 and 10, i.e., of preschool and primary school age. Later, a computer version was developed [5].

From the earliest age, children hear and read fairytales. In a fairytale characters embody certain ideologemes shared among adults and transmitted to a child through social training. A fairytale constitutes a “model” text that imparts social norms [6]. Fairytales convey certain norms and rules from social to individual consciousness. Social consciousness contains the norms and traditions of a group with a distinct cultural and historical identity that determines its system of values. The role of critical reflection in this process of norm acquisition, if any, is typically inessential [7].

Fairytales provide children with a framework for understanding the difference between “good” (the spectrum of positive values and characteristics) and “evil” (the spectrum of negative values and characteristics), as well as with the rules of proper conduct in various contexts.

By analyzing a child’s attitude to a certain fairytale character, it is possible to identify his or her moral values.

Through identification with characters, a child develops his/her behavioral guidelines. The attribution (understanding) of the personality qualities of a fairytale character is an important stage in the development of interpersonal perception, which is essential in the social life of a person.

The psychosemantic method combines the capabilities of qualitative methods (for example, projective—the identification of “deep”, non-reflective, implicit content) with the objectivity of quantitative research methods, which is very important in the study of children. In particular, it allows researchers to get a reliable picture of the cultural characteristics of mental development. However, the research capabilities of such a method need empirical evidence. The first use of FSD [1] was performed on adults; it showed the possibility of constructing the semantic space of fairytale characters and combining images of fairytale characters and living people in one semantic space. Over the next quarter-century, FSD was used for individual counseling, as a case study application for selective research studying child development limited to a few unpublished student diplomas on small samples of children. The need arose to conduct a full-scale study to empirically substantiate the reliability and validity of the psychosemantic method, which allows diagnosing the state of the cognitive sphere of interpersonal perception of other people and self-perception. As part of this task, it was proposed to investigate the possibility of revealing the cultural characteristics of the development of children living in different countries within the same language space, differing in the traditions of the approach to education, to compare the obtained data with the age, gender, and cultural differences established in developmental psychology.

## 2. Method

During the survey session, the participant evaluated a fixed list of fairy characters by a fixed list of personal traits, which were formulated in terms that child respondents were familiar with, i.e., words used by parents or preschool and school teachers when communicating with children of this age. List of characteristics: *Loyal friend, Brave, Beautiful, Kind, Sly, Greedy, Crybaby, Clever, Snitch, Well-behaved, Boastful, Skillful, Bully, Naughty, Cheerful.* List of characters in the basic version of FSD: *Aibolit, Buratino, Puss in Boots, The Snow Queen, Karabas-Barabas, Karlsson, Malvina, Pierrot* [5]. If the child was not familiar with a character, it was replaced in the assessment list of characters with a more popular character with similar personality traits.

If the child agreed that a character possessed a certain trait, his/her response was coded as 1; disagreement was coded as −1. The question could also be evaded by choosing a “Somewhat so” answer. In this case, the response was coded as 0.

Along with fairytale characters, the child assessed himself/herself and could also additionally assess significant adults (parents, kindergarten, or school teachers) and peers.

The child’s answers were grouped into a data matrix (characters’ scores on the primary variables—personal characteristics).

The results can be interpreted in a variety of ways.

It is interesting to look at the correlations between these personal characteristics in a child’s representations. For example, among boys, the variable *Crybaby* most often negatively correlates with *Loyal friend* and *Bold*, while among girls negative correlations are with *Evil* and *Rude*. It is therefore possible to determine how personal correlations correspond to tendencies reflecting belonging to a particular gender or subculture group (determined, for example, by a child’s ethnicity). In addition, the resultant data matrix was processed using principal component analysis. The content of the components enables the researcher to understand the individual properties of personal constructs. The percentage of the variance of each component establishes its hierarchy. The analysis of a child’s semantic space, where the fairytale characters and the position of the child (“myself”) are located in the form of coordinate dots, allows personal counselors to reconstruct a fragment of the child’s worldview and glimpse the world through the child’s eyes.

A self-evaluation measure was introduced by assessing “myself” according to the primary variables and comparing the positions of “myself” and other characters.

The position of the child him/herself (coordinates of the dot corresponding to self-assessments in the semantic space) demonstrates the specifics of self-awareness and identification (which characters he/she is psychologically closer to). The closer the point corresponding to “myself” is to the point of a certain character, the stronger the identification with this character. This identification (or distance) helps to interpret the child’s self-esteem [5].

By analyzing the positions of additional personages and identifying the degree of their closeness to the positions of fairytale characters it is possible to determine the nature of the child’s attitude to a certain adult. For example, a kindergarten teacher’s semantic closeness to the character of *The Snow Queen* [8] or *Baba Yaga* (a character from Russian folklore, a fearsome old witch [9]) can be a warning sign for both parents and the teacher.

Moreover, several indicators can be used to compare individual results among children. These indicators can provide some psychological insights; they can be compared with the same indicators in other children and thus be used to determine age norms.

### 2.1. Cognitive Complexity of Interpersonal Perception 

Cognitive complexity of interpersonal perception is one such indicator. It is one of the main indicators of individual development. The simplest form of categorization in the FSD technique is one-dimensional. All positive traits form one pole of this category, while the other pole is represented by negative traits.

If a child is not sufficiently developed, e.g., is still small, or has a low mental age due to a mental disorder, then a lot of personal qualities for him/her are glued together based on an evaluation component.

So, if a character is kind, for example, *Ouch* (*Aibolit*) (the central character of a series of children’s books by K. Choukovsky [10], loosely based on *Doctor Dolittle* created by Hugh Lofting [11]), then from the point of view of a cognitively simple person he is beautiful and well-behaved, while *The Snow Queen* [8] is evil, ugly, and stupid. Individual characteristics, thus, for an undeveloped child are highly correlated with each other. However, in the course of development, a child can begin to understand that a character, for example, *Cheburashka* (a character in a popular Soviet cartoon by E. Uspenky [12]), can be kind, but not too beautiful, and the evil *Snow Queen* can be called beautiful. In other words, in the course of development, the semantic differentiation of the personal qualities attributed to the characters takes place, and they are split into several discrete characteristics.

In FSD cognitive complexity is determined in several ways [5]. Here we used the following formula [13]:

(1) $$CC=1\;-\;\sigma_{1}^{2};\;\sigma_{1}^{2}\;\in \;[0;\;1],\;$$

where $\sigma_{1}^{2}$ is the percentage of contribution of the first factor in a non-rotated solution to the total variance. In psychological terms, this indicator demonstrates that cognitively simple individuals have a less dimensional space, i.e., most items load highly on the first component and its contribution to the total variance is significantly higher compared to other components, with no significant loadings on other components. In an extreme example, there is only one component, e.g., “good/bad”, “like/dislike”, and its contribution to the total variance approximates 100%. In this case $\sigma_{1}^{2}=1$. In contrast, more cognitively complex individuals have more categories of perception and their contributions to the total variance are more evenly distributed, which decreases the contribution of the first component. It should be noted that here cognitive complexity refers to the domain of interpersonal perception and a higher level of its development is not necessarily linked with academic intelligence.

### 2.2. Measurement of Socialization

Socialization is a process through which a child acquires norms and values shared by the adults in his or her environment.

To assess the level of child socialization, we compared two data sets: the child’s response matrix and the “normative” matrix (the averaged matrix of adult responses). A measure of socialization of the child is the degree to which his/her assessments of the characters are similar to adults’ assessment.

As a result, socialization was calculated as follows. For a set of estimates {*o_ij_*}, the matrix of the estimates provided by the child, and the adult matrix *E* = {*e_ij_*}, obtained by averaging the ratings of each character for each scale in the adult sample, we calculated the socialization index with the Pearson formula:

*Soc* = (*r*({*o_ij_*}, {*e_ij_*}) + 1)/2, *i* = 1…*M*; *j* = 1…*N*, (2)

where *N* is the number of primary variables, and *M* the number of personages.

The index is lower than 1. The higher the socialization, the closer the value is to 1, and the more the matrix of the child respondent resembles the “adult” matrix; therefore, the child can be said to have learned the normative rules for evaluating characters according to the evaluation characteristics, as they are “accepted” among adults.

Cognitive complexity and socialization should be interpreted together. For example, children with schizophrenia can demonstrate high cognitive complexity (primary items are independent (low-correlated)), but the ratings of the characters themselves are strikingly different from the adults’ ratings and thus show that this child has a low measure of socialization.

### 2.3. Participants

The respondents were children aged 4–10 years old, attending kindergarten or primary school in Russia, Azerbaijan, Uzbekistan (all Russian-speaking), totaling 1205 subjects. Each participant performed the procedure individually in the presence of the experimenter. The parents or representatives of all subjects provided their informed consent for inclusion before participating in the study. The study was conducted in accordance with the Declaration of Helsinki, and the protocol was approved by the Ethics Committee of the Faculty of Psychology of Lomonosov Moscow State University (Project No. 2016/42).

## 3. Results

### 3.1. Using FSD for Individual Work (“Case Study” Results)

FSD can be used either for individual counseling work which a psychologist can lead with a child or in screening studies where the method can be used for getting sample data. Here, we take a look at the results of the analysis of an individual child’s answers. The data were analyzed by the principal component method with varimax rotation.

**Figure 1:**
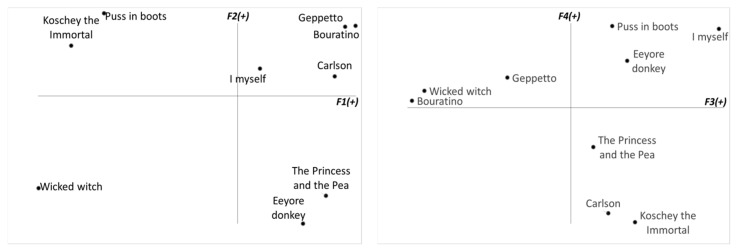
Semantic space of respondent *N*.

The respondent (Figure 1, Table 1) was a four-year-old boy from the Russian sample. His cognitive complexity was high and socialization was moderate. We can see that the boy has a high level of self-criticism. His positions on factor 1 and factor 2 were not very high, but at the same time he accepted himself, and his evaluations on factors 3 and 4 were the highest. It should be noted that while factors 1, 2, and 4 on the right poles had positive traits only, the third factor was ambivalent and included not only the positive traits *Well-behaved* and *Clever* but also negative traits such as *Boastful* and *Crybaby.* This fact confirms the results of the cognitive complexity calculation.

During the individual session, the psychologist should pay attention to spontaneous comments that children make in response to the FSD questions. They can be very helpful in interpreting the quantitative results. Below are some examples of such comments that were obtained from Russian respondents.

A seven-year-old boy: “I’ve read about Uncle Styopa with my mum. I’ve seen a cartoon about Buratino. Oh!!! Signor Tomato is from the fairytale about Cipollino. I’ve seen the cartoon only once. He is a best friend that never lets you down. ‘Beautiful’ is probably about Malvina. I think I’m also a bit beautiful but I have freckles. One person thinks I’m beautiful—that’s my friend.” Responding to the psychologist’s comment that he looked good with freckles, he said “Do you think so?” and smiled. “I’m probably also a little greedy, I’m not always willing to share. And a kind person is the one who shares everything.”

A six-year-old girl: “’Sly’ is like a fox. A kind one doesn’t let you down. Beautiful, this is like Malvina. She has beautiful hair and face. I’m beautiful too. ‘Skillful’ is good with his hands. For example, I can draw and make plasticine models and different collages. My grandma says I’m skillful.”

A five-year-old girl: Described the Snow Queen as unkind because she was mean to Kai. “Doctor Aybolit is kind, he felt sorry for all the animals, he treated them. ‘Sly’ is like a sly fox. ‘Well-behaved’ says ‘hello’ and ‘thank you’ and also always does the right thing. A snitch says about everyone that they’ve done something.” 

A five-year-old boy: “It’s good to be sly, you can’t be sly and silly, can you? Puss in Boots is anything but silly. He got his owner a palace. He tricked everyone and managed to trick the lion into turning into a mouse, and then ate it. Or the lion could have eaten Puss in Boots. Matroskin the Cat is also sly and clever. He got a cow.”

A six-year-old boy: “Doctor Aybolit isn’t clever, is he? He treats animals for free. But he is kind, he never abandons them. He feels sorry for them. Puss in Boots is the clever one. His owner didn’t have anything, he was poor. But Puss got him everything: clothes, a palace. And Puss is also brave. He wasn’t afraid of the lion.”

A five-year-old girl: “Aybolit—I know this one. It’s a fairytale by Chukovsky. I’ve read it with my mum. The Snow Queen, I’ve seen a movie about her. She stole Kai and froze his heart. [After that] he doesn’t love anyone. He used to love Gerda but then stopped. “Clever” is the one who thinks all the time, it’s good to be clever. For example, Buratino isn’t clever, he buried the money in the ground and thought that they would grow. Money … you need to go to work to have it. You need to earn it. Doctor Aybolit is clever, he’s an animal doctor. You need to study a lot to become a doctor.”

A four-year-old girl: “’Well-behaved’ always does the right thing and teaches others to be good. I do everything right. I say ‘thank you’ and tidy away my toys. I’ve got a kitten, Murzik. I teach him, tell him where his litter box is. He is too small for now … he doesn’t understand. I don’t bully him. Karabas Barabas is ill-behaved, he was nasty to the actors. They wanted to run away from him.”

### 3.2. Using the FSD Method for the Comparative Empirical Study

As mentioned earlier, the FSD can also be used as a group test. In such cases the method is used for screening research. Indices of cognitive complexity of interpersonal perception, socialization, self-esteem, and others can be used for drawing conclusions about the sample, uniting the respondents who participated in the study as a whole.

The cross-cultural study was conducted in former Soviet republics: Russia (mainly Moscow), Azerbaijan (mainly Baku), and Uzbekistan (mainly Tashkent). The respondents from all three countries were Russian-speaking. The cultural environments, however, are drastically different. Azerbaijani and Uzbek children do not know the fairytales that Russian children hear and read. As a result, while the same set of assessment characteristics was used in all three samples, the characters had to be replaced [14]. Table 2 shows the sample composition for each of the three countries.

Since the objects (characters) evaluated by the respondents in the Russian sample were different from those evaluated in Baku and Tashkent, we decided it would be incorrect to compare raw results among the three samples. As a result, it was more important to compare the relative age dynamics of these indices and the relative differences between boys and girls in each age group. To this end, each sample variable for ***CC*** and ***Soc*** (see Equations (1) and (2)) was transformed to a Z-score.

**Figure 2:**
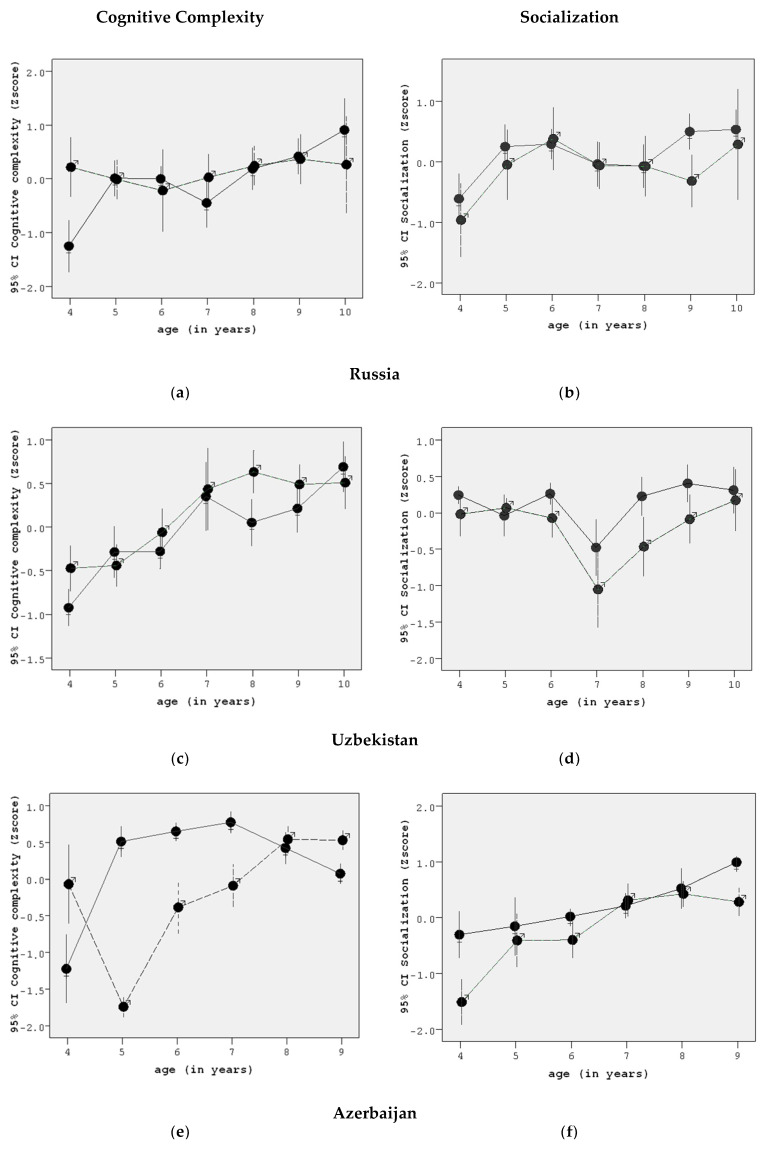
Age dynamics of cognitive complexity and socialization in each sample. Girls—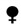; Boys—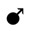. (**a**) the age dynamics of cognitive complexity for boys and girls in Russia; (**b**) the age dynamics of socialization for boys and girls in Russia; (**c**) the age dynamics of cognitive complexity for boys and girls in Uzbekistan; (**d**) the age dynamics of socialization for boys and girls in Uzbekistan; (**e**) the age dynamics of cognitive complexity for boys and girls in Azerbaijan; (**f**) the age dynamics of socialization for boys and girls in Azerbaijan.

The graphs in Figure 2 demonstrate the age dynamics of cognitive complexity and socialization for boys and girls in three samples. Figure 2 presents the means and 95% confidence intervals for each sex and age group. Such visualization makes it easy to see if the mean in one subsample significantly differs from the mean for another subsample. In such cases the intersection of two confidence intervals of these subsamples is empty.

## 4. Discussion

In all three samples, we can see that both indices increase with age. However, we should note several abrupt lows. For instance, the decrease in socialization in the Tashkent sample at the age of 7 can be explained by the fact that children start school at this age: a child now has a new significant adult (the teacher)—along with his/her parents—with a system of values which can be quite different from the family one and thus the child is faced with a confusing choice.

In Moscow, seven-year-old girls demonstrated some decrease in cognitive complexity when starting school, which can be interpreted as an attempt to organize one’s perceptions to adapt more easily to new school requirements. Teachers prefer obedient and thus more cognitively simple children, as they are easier to handle, and girls match teachers’ expectations to a higher degree.

Another common phenomenon is the significant difference in cognitive complexity between girls and boys at the age of four. This index is higher for boys. However, a comparison with the socialization indices (which are lower among boys) demonstrates that a higher index of cognitive complexity is more likely to be a result of the underdevelopment of cognitive structures leading to random answers, rather than a higher level of development. By the age of 5 years, boys develop cognitive structures that can overcome the chaotic nature of responses provided by four-year-olds. This abrupt decrease in cognitive complexity combined with a higher socialization index is especially evident in the Azerbaijani sample.

Boys did not score higher socialization indices in any age group, which may suggest that girls are more adaptive to new circumstances. This may be related to the fact that girls tend to be more communicative. Good people skills are regarded as a necessary quality for a woman in traditional society, with women being responsible for nurturing children, creating a positive atmosphere at home, etc., while men are seen as protectors and breadwinners, which emphasizes the qualities of being smart and strong [15].

Another noticeable feature is a significant drop in socialization at the age of seven, both for girls and boys. This is the age when, by starting school for the first time, children enter “adult” life and expand their social environment [16,17]. Stepping out of the familiar social (family) environment can explain some disorientation.

Boys and girls develop differently and in practically all cases we can see that girls tend to be ahead in the cognitive development of the interpersonal sphere by approximately a year and that this development is typically more evenly paced (e.g., instance 2f).

A finding that stands out is that ten-year-olds showed no clear progress in socialization compared to five-year-olds. This demonstrates that fairytale characters are already “processed” at an earlier age, and that in older children, the level of socialization should be assessed with fictional characters (for example, from books by Ivan Turgenev, Jack London, etc.) that define the zone of proximal development for children of this age.

## 5. Conclusions

This article presents the possibilities of using a Fairytale Semantic Differential method for working with children 4–10 years old. This technique was developed in Russia but can be easily adapted to the work of a psychologist with respondents living in other countries of the Russian-speaking space. The method makes up for the shortage of objective psychodiagnostic methods for young children.

We expect this psychosemantic technique to come into wide use for the diagnostic assessment of preschool and primary school children including personal counseling.

This article presents both the results for an individual subject (“case study” results) and the results of a cross-cultural empirical study including data from three different samples aimed at comparing developmental trajectories of cognitive complexity and socialization in three countries. These three countries share a common Soviet past, but the vectors of post-Soviet development have led to quite significant cultural differences, including gender-specific child-rearing and their attitude to entering adult life, particularly school. This comparative study was possible due to a common language.

Together with the identified trends in the cognitive development of interpersonal perception, the results in the figures clearly demonstrate the differences.

The identified age and sex differences in the indices are in line with expectations. Cognitive complexity and socialization demonstrate a positive development with age and are different for boys and girls. Notably, these indices show nonlinear age dynamics, which, from our point of view, correspond to the non-linearity of cognitive development in childhood. Girls somewhat outpace boys in cognitive aspects related to the development of social intelligence.

The empirical evidence for the decline in self-esteem and socialization that occurs when a child starts school points to an age crisis and demonstrates that psychological and pedagogical support is essential during the adaptation period when children enter school life. In societies where family education implies long-term support and provides a “buffer” in child–school interaction (e.g., Azerbaijan), changes in cognitive indices at the time of starting school are more gradual.

In our study, we do not claim to have received new results about the dependence of cognitive development on the style of raising families. We used this knowledge, known in psychology, to explain the obtained differences in cognitive development in different cultures, which differ, including the most common family raising practices. Similarly, we are not claiming to be the first to make the statement that girls’ higher adaptability to new circumstances may be related to the fact that girls tend to be more communicative, but we used this knowledge to explain the gender differences in socialization obtained in our study. On the whole, the results we obtained with the help of psychosemantic research are consistent with the facts known in developmental psychology, confirming the validity of our Fairytale Semantic Differential method.

Given the fact that more and more children in former Soviet republics stop speaking Russian (especially those living outside of capitals and large cities), it would be interesting to compare the results of surveys conducted in different subcultures of one country. This would require developing a new set of fairytale characters that are familiar to child respondents as well as translating the list of 15 characteristics. The results of this study allow us to confidently expect success in such comparative studies in the future.

The use of the Fairytale Semantic Differential in school and pre-school educational institutions will facilitate the preliminary work of psychologists of these institutions to identify children with problems, including problems with relationships with significant adults (parents, teachers) and peers. The limitations of this method are connected with the fact that in order to use it, the acquaintance of the child with the fairytale characters included in the list for assessment is required. That is why one of the directions for improving the method is expanding the set of characters that can be offered to a child for evaluation.

## Figures and Tables

**Table 1:** Factors: loadings and total variance (respondent *N*).

% of Variance	F1 (+)	Loadings	F1 (−)	Loadings
	Beautiful	0.95	Sly	−0.97
32.284	Loyal friend	0.95		
	Kind	0.93		
	**F2 (+)**		**F2 (−)**	
20.197	Cheerful	0.94	Snitch	−0.96
	Brave	0.60		
	**F3 (+)**		**F3 (−)**	
18.274	Well-behaved	0.78	Bully	−0.65
	Boastful	0.74		
	Clever	0.70		
	Crybaby	0.60		
	**F4 (+)**		**F4 (−)**	
16.633	Skillful	0.94	Greedy	−0.70
			Naughty	−0.59

**Table 2:** Sample distributions by age and sex.

		Russia			Uzbekistan			Azerbaijan		
	Sex	Girls	Boys	Total	Girls	Boys	Total	Girls	Boys	Total
Age										
4		21	14	35	50	50	100	25	25	50
5		36	23	59	50	50	100	25	25	50
6		40	12	52	50	50	100	25	25	50
7		23	21	44	34	23	57	25	25	50
8		29	24	53	53	51	104	25	25	50
9		15	21	38	45	42	87	25	25	50
10		8	7	15	25	36	61			
Total		174	122	296	307	302	609	150	150	300
